# Metabolic alterations and the potential for targeting metabolic pathways in the treatment of multiple myeloma

**DOI:** 10.20517/2394-4722.2019.05

**Published:** 2019-04-03

**Authors:** Dustin Rizzieri, Barry Paul, Yubin Kang

**Affiliations:** Division of Hematological Malignancies and Cellular Therapy, Duke University Medical Center, Durham, NC 27710, USA.

**Keywords:** Metabolism, alterations, multiple myeloma, treatment

## Abstract

Metabolism is defined as the collection of complex biochemical processes that living cells use to generate energy and maintain their growth and survival. Metabolism encompasses the synthesis and breakdown of glucose, fatty acids, and amino acids; the generation of energy (ATP); and oxidative phosphorylation. In cancer cells, metabolism can be commandeered to promote tumor growth and cellular proliferation. These alterations in metabolism have emerged as an additional hallmark of various cancers. In this review we focus on metabolic alterations in multiple myeloma (MM) - a malignancy of plasma cells - including derangements in glycolysis, gluconeogenesis, the tricarboxylic acid cycle, oxidative phosphorylation, and fatty acid/amino acid synthesis and degradation. Particular focus is given to metabolic alterations that contribute to myeloma cell growth, proliferation and drug resistance. Finally, novel approaches that target metabolic pathways for the treatment of MM are discussed.

## MULTIPLE MYELOMA

Multiple myeloma (MM) is a malignancy of terminally differentiated plasma cells typically characterized by clonal proliferation of these plasma cells in the bone marrow. MM represents 1% of all malignancies and 18% of hematologic malignancies in the United States; accounting for an estimated 30,770 new diagnoses and 12,770 deaths in 2018 alone^[1]^. Classically, MM results in the secretion of a non-functional monoclonal immunoglobulin (Ig) produced by the transformed plasma cells. Production of this aberrant Ig results in several of the complications associated with MM such as renal dysfunction, neuropathy, and hyperviscosity syndrome^[2,3]^. However, in approximately 15%-20% of patients the abnormal plasma cells secrete only monoclonal free light chains, and in approximately 2%-3% of cases these cells secrete no monoclonal protein at all resulting in the so-called non-secretory myeloma^[4,5]^. Myeloma cell growth in the bone marrow and the resultant cytokines produced by these transformed cells and/or other cells in the bone marrow microenvironment lead to the classic symptoms of active MM: osteolytic bone lesions, hypercalcemia, and anemia^[6]^.

The underlying epidemiology of MM remains largely undefined. Previously, exposure to ionizing radiation was thought to be a risk factor, but this was subsequently refuted in a large cohort of atomic bomb survivors in Japan^[7]^. More recent data suggest a 2-3 fold increased risk for development of MM among African Americans which is thought to be related to increased rates of MGUS among this population^[8,9]^. Interestingly, in contrast to most other malignancies, African Americans with MM tend to have a better prognosis compared to age-matched Caucasians with the disorder^[10]^. Several meta-analyses have suggested obesity is associated with increased risk of myeloma with a relative risk ranging from 1.11-1.82^[11-14]^, and it has been shown that obesity significantly increases the risk of myeloma associated mortality^[15-16]^.

MM is primarily a disease of the elderly, with the median age at diagnosis being 69 in the United States^[1]^. This population often suffers from significant co-morbidities making management of myeloma more challenging. Specifically, approximately 40% of this population meets criteria for obesity (BMI ≥ 30)^[17]^, and the rates of several obesity associated metabolic disorders such as diabetes and hyperlipidemia already approach 25% and 50% respectively, and continue to rise^[18,19]^. Given these associations it is reasonable to wonder if these metabolic changes are significantly participating in MM pathogenesis. As this area has largely been uncharacterized, in this review we aim to highlight the metabolic changes that occur in myeloma patients, summarize how these changes are affected by myeloma directed therapy, and suggest possible interventions to enhance anti-myeloma based therapies by taking advantage of metabolic pathways which are often dysregulated in MM patients.

## OVERVIEW OF METABOLISM

### Glycolysis

Glycolysis is the primary process by which cells break down glucose releasing stored energy in the process which can be used to generate ATP^[20]^. This process begins when membrane bound insulin receptors bind insulin resulting in autophosphorylation of the tyrosine residues. Subsequent phosphorylation of insulin receptor substrates, and activation of the PI3K and MAPK pathways promote cellular uptake of glucose^[21]^. Insulin also regulates fructose 2,6-bisphosphate, a key regulator of glycolysis. In glycolysis, a single glucose molecule is phosphorylated by hexokinase (HK) to yield glucose-6-phosphate (a reaction which requires ATP), which then undergoes isomeric change by the enzyme glucose 6-phosphate isomerase to become fructose 6-phosphate. Fructose 6-phosphate is then irreversibly phosphorylated by phosphofructokinase (PFK) in a reaction that requires ATP to yield fructose 1,6-bisphosphate; this reaction is the rate-limiting and committed step of glycolysis. Fructose 1,6-bisphosphate is then cleaved by aldolase into two triose phosphates: glycerolaldehyde-3-phosphate and dihydroxyacetone phosphate. Glycerolaldehyde 3-phosphate is oxidized by glycerolaldehyde 3-phosphate dehydrogenase using NAD+ as an electron donor to yield 1,3-bisphosphoglycerate (1,3-BPG). 1,3-BPG is then dephosphorylated at carbon-1 by phosphoglycerate kinase to yield 3-phosphoglycerate, and then reversibly converted to 2-phosphoglycerate by phosphoglycerate mutase. 2- phosphoglycerate undergoes a dehydration reaction catalyzed by enolase to form phosphoenolpyruvate (PEP) which is then irreversibly converted to pyruvate by pyruvate kinase (PK) in a reaction that also generates ATP^[22,23]^.

In eukaryotic cells, pyruvate can be reduced to produce ATP through two different pathways depending on the presence of mitochondria and appropriate blood and oxygen supply in the tissue of need: aerobic respiration and anaerobic respiration. In aerobic respiration, pyruvate is transported into the mitochondria through a specific transporter and then decarboxylated by pyruvate dehydrogenase to produce acetyl-CoA which serves as the initial substrate for the tricarboxylic acid (TCA) cycle (see section 2 below). In anaerobic respiration, which in eukaryotes is typically limited to cells without mitochondria or poorly vascularized tissue (either endogenous or induced by states of physiologic stress), pyruvate accepts a hydrogen from NADH to produce lactate and NAD+. The NAD+ produced can then be used for glycolysis reactions or even react with TCA intermediates. Energy yield from anaerobic glycolysis is significantly reduced compared to its aerobic counterpart, however this process is more rapid and can occur in oxygen deprived tissues^[22]^. Notably, some cancerous cells have been shown to preferentially undergo anaerobic respiration even under optimal conditions for aerobic respiration^[24-26]^. This effect, which is thought to be due to metabolic reprograming related to their hyperproliferative state, has been termed the “Warburg effect” after German physiologist and Nobel laureate Otto Warburg whose initial observations of increased glucose consumption coupled with increased lactate excretion from cancer cells led to the hypothesis of its existence^[27-29]^.

Cells have developed several mechanisms to increase or decrease glycolysis in response to metabolic needs. Furthermore, metabolic control of glycolysis occurs through various feedback mechanisms. Inhibition of insulin receptor signaling or key enzymes in the pathway (especially PFK) typically occur in fasting states or high energy states when the ratio of ATP to ADP is high. In these situations metabolism is shifted away from glycolysis and can instead result in a reciprocal induction of gluconeogenesis (see section 4 below)^[22,23]^. Typically, glycolysis follows the general steps as reviewed above. However, there is some redundancy in the metabolic processes of the cell and some molecules may be synthesized in a different way and still be used in glycolysis. This can be seen in the way in which cells breakdown disaccharides like sucrose. If energy is needed, then the sucrose will be converted into fructose-6-phosphate molecules and continue through glycolysis^[22]^.

### The TCA cycle

The TCA cycle allows for complete catabolism of organic molecules in the presence of oxygen, and results in the majority of ATP production in eukaryotes. The reactions of the TCA allow for breakdown of carbohydrates as well amino acids and fatty acids through various metabolites that can enter the pathway at different steps. For glucose metabolism the process begins with the production of acetyl Co-A in the mitochondrial matrix through oxidative decarboxylation of pyruvate in a reaction catalyzed by the pyruvate dehydrogenase complex (a complex of 3 component enzymes, 2 regulatory enzymes, and 5 coenzymes). Acetyl Co-A and oxaloacetate combine to form citrate in a condensation reaction catalyzed by citrate synthase. Citrate is then isomerized to isocitrate by aconitase which is subsequently converted to a-ketoglutarate by oxidative decarboxylation in a reaction catalyzed by isocitrate dehydrogenase. The a-ketoglutarate dehydrogenase complex catalyzes the conversion of a-ketoglutarate to succinyl Co-A and producing NADH. Succinyl Co-A is then cleaved to succinate by succinate thiokinase in a reaction that generates GTP via substrate level phosphorylation. Succinate is subsequently oxidized to fumarate by succinate dehydrogenase with FAD being reduced to FADH_2_ in the process. Fumarate is then hydrated to malate in a reaction catalyzed by fumarase, with malate being oxidized to oxaloacetate by malate dehydrogenase producing another molecule NADH in the process.

There are many ways that the TCA cycle is regulated. The first regulatory step is known as the bridge reaction where pyruvate is converted to acetyl-CoA. If the cell has too many high energy molecules, then this “bridge reaction” will not occur and pyruvate will be utilized in other fashions. Other regulatory steps include the synthesis of citrate, and the oxidative decarboxylations of isocitrate and a-ketoglutarate all of which result in the production of high energy molecules (NADH, FADH2, ATP, GTP) which then undergo oxidative phosphorylation. These steps will be inhibited if the concentration of the high energy molecules is increased. Similarly, if the concentration of low energy molecules (GDP, ADP, NAD+, FAD) were to accumulate then the reactions in the TCA cycle would increase^[22]^.

### Oxidative Phosphorylation and the electron transport chain

Many of the products from glycolysis are then transported to the inner mitochondrial membrane where they donate electrons to a set of electron carriers known as the electron transport chain (ETC). As electrons transverse the ETC they lose energy and are coupled to the pumping of protons across the inner mitochondrial membrane which can subsequently be captured to drive the production of ATP through oxidative phosphorylation (OXPHOS) in a reaction catalyzed by the enzyme complex ATP synthase^[22,23]^.

Oxidative phosphorylation is regulated via several mechanisms. The enzymes succinate dehydrogenase and α-ketoglutarate dehydrogenase have roles in both OXPHOS and the TCA cycle, and are regulated by TCA intermediates like succinate, fumarate, and α-ketoglutarate. These two enzymes are also inhibited by high energy compounds and activated by low energy compounds. The pH of the mitochondrial matrix also regulates OXPHOS, as the ability to maintain the ETC and the subsequent proton pump is tied to maintaining a pH gradient^[22]^.

### Gluconeogenesis

Gluconeogenesis is the synthesis of glucose, allowing the body to ensure blood sugar levels remain stable even in fasting states. The first step in gluconeogenesis is synthesizing PEP from pyruvate. This is a two-step process in which pyruvate is first converted to oxaloacetate by pyruvate carboxylase, then converted to PEP by PEP carboxykinase. PEP is converted to 2-phosphoglycerate, and subsequently to 3-phosphoglycerate, which is phosphorylated to produce 1,2-bisphosphoglycerate. Glyceraldehyde 3-phosphate is then synthesized by glyceraldehyde phosphatase dehydrogenase and converted to fructose 1,6-bisphosphate. Finally, glucose 6-phosphatase dephosphorylates glucose 6-phosphate to form glucose. Since this process is nearly the opposite of glycolysis, many of the enzymes between these two processes are the same, and gluconeogenesis is tightly regulated by many feedback mechanisms to maintain the glucose concentration and to avoid hypo/ hyperglycemia. Not surprisingly then, one of the main regulators of gluconeogenesis is glucose itself; the other two main regulators are pyruvate and PEP. High concentrations of these two molecules in combination with lower levels of glucose leads to increased gluconeogenesis^[22]^.

### Fatty acid synthesis and degradation

Fatty acid synthesis is not only important for long-term energy storage but also for structural components of cell membranes and eicosanoids. Fatty acid synthesis typically begins with a reaction between acetyl-CoA and malonyl-ACP to yield acetoacetyl-ACP and C0_2_. This condensation reaction is facilitated by 3-ketoacyl ACP synthase (KAS III). Acetoacetyl-ACP subsequently undergoes reduction, hydration, and re-reduction to reduce the 3-keto group eventually yielding acyl-ACP, which can be used to initiate elongation of the fatty acid chain. This cycle continues until acyl-ACP’s backbone reaches 16 or 18 carbons. After appropriate elongation acyl-CoA can be used for multiple different processes, including synthesis of glycerophospholipids and triacylglycerides, production of phosphatidate, and synthesis of phosphatidylcholine (PC). While lipogenesis can occur through the conversion of carbohydrates to acetyl-CoA, it can also take place by *denovo* lipogenesis through the conversion of glycogen to fatty acids which typically occurs when glycogen storage is full^[22]^.

To release energy stored at fat, the molecule undergoes β-oxidation, or fatty acid oxidation. As fatty acids are unable to diffuse through the mitochondrial membrane, they are first converted to acylcarnitine which can enter through the carnitine antiports. Once in the matrix, acylcarnitine is converted to fatty-acyl-CoA. β-oxidation is largely the reverse reaction of lipid synthesis. Starting with acyl-SCoA there is oxidation, hydration and oxidation again to yield 3-ketoacyl-SCoA. The β-carbonyl is then cleaved by HS-CoA, resulting in a fatty Acyl-CoA molecule that now holds two less carbons then it did at the start of the cycle. Each cycle thus produces ubiquinol, NADH and acetyl-CoA which can all be used in aerobic respiration^[22]^.

Fatty acid synthesis and degradation are regulated largely by cellular energy dependence. Fatty acids serve as long-term energy storage molecules. During starvation where ATP production from the breakdown of glycogen cannot produce adequate amounts of energy, fatty acid degradation accelerates. On the other hand, if there are adequate supplies of ATP and glycogen storage is full, then fatty acid synthesis can occur^[23]^.

### Amino acid synthesis and degradation

Amino acids and proteins remain a central aspect of cellular metabolism. While there have been over 300 amino acids described, only 20 are commonly found in mammalian proteins. Not surprisingly, these 20 amino acids are the only amino acids coded for by DNA^[30]^. Of these, histidine, isoleucine, leucine, lysine, methionine, phenylalanine, threonine, tryptophan, and valine are termed essential as they cannot be synthesized by humans. Amino acids serve various roles in metabolism. In addition to protein synthesis, they can be used for energy production and synthesis of hormones. In extreme situations where energy is scarce and fatty acid reserves have been exhausted, protein turnover becomes a main source of energy^[22]^.

Oxidation of proteins typically occurs in small quantities since the ammonia/ammonium byproduct is toxic and must be transported bound to *L*-glutamine. *L*-glutamine can leave the tissue and be transported to the liver for energy production with NH4+ excreted as urea. Alternatively, proteins can be metabolized by the glucose-alanine cycle. When breakdown of muscle protein yields *L*-glutamate, a reaction with pyruvate occurs yielding alanine and α-ketoglutarate. Alanine is then transported to the liver where transamination occurs resulting in glutamate which subsequently undergoes deamination releasing NH4+ into the urea cycle. Pyruvate is also a byproduct of the transamination reaction and can be used in the liver for gluconeogenesis.

### Integration of metabolic processes

Cellular metabolism is intertwined and tightly regulated. Glycolysis and the TCA cycle produce many byproducts that may enter the ETC for oxidative phosphorylation or can be used for other purposes. For example, the oxidation of glyceraldehyde 3-phosphate which occurs in glycolysis also results in the production of NADH and H+. These molecules can serve as electron donors for the ETC. Similarly, FADH2 which is a byproduct of the reaction that converts succinate to fumarate in the TCA cycle can also serve as an electron donor in the ETC.

Anaerobic and aerobic respiration are closely linked by the use of NAD+ and NADH. Production of lactate during anerobic respiration is one of the main ways in which NADH and H+ are oxidized back into NAD+ which allows for the TCA cycle to continue without any significant changes. Ultimately, glycolysis, the TCA cycle, gluconeogenesis, fatty acid metabolism, and protein synthesis are all interconnected and exist in a highly regulated and interdependent environment designed to maintain homeostasis^[31-33]^.

## ALTERATIONS IN METABOLISM OF MULTIPLE MYELOMA

**Figure 1.**
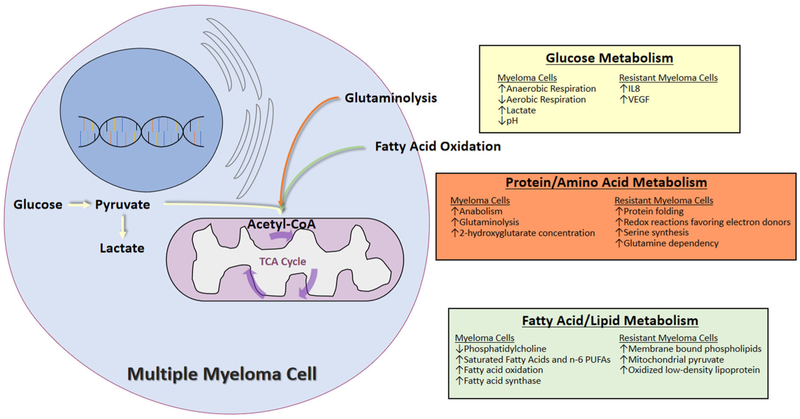
Summary of the metabolic changes in multiple myeloma cells and in resistant myeloma cells

Multiple changes in metabolism occur in myeloma cells which are summarized below and in Figure 1. Understanding the metabolic alterations in MM has important implications in the care of patients with MM. It sheds new light into our understanding of MM development and progression, and allows for the assessment of predictive or prognostic biomarkers for optimal management of myeloma patients. Importantly, it provides potentially novel therapeutic targets for the treatment of MM.

Over the last decade, several technologies such as mass spectrometry, ^1^H-nuclear magnetic resonance (^1^-NMR) spectroscopy and mass spectrometry, and ^13^C stable isotope resolved metabolomics (SIRM) have been used to determine metabolic alterations in monoclonal gammopathy of undetermined significance (MGUS) patients, newly diagnosed MM (NDMM) and relapsed/refractory MM (RRMM) and to compare metabolic changes in drug resistant MM cells. Steiner *et al*.^[34]^used mass spectrometry to analyze 188 endogenous metabolites in peripheral blood plasma samples of healthy controls, patients with MGUS, NDMM, or RRMM. The metabolomic profile was quite different between healthy controls and patients with MGUS, NDMM or RRMM with significant alterations in amino acid, lipid and energy metabolism. Eight plasma metabolites - free carnitine, acetylcarnitine, glutamate, asymmetric dimethylarginine (ADMA) and four PC species - were different between MGUS and NDMM patients, demonstrating that metabolic changes occur during MM development and progression^[34]^.

High-resolution ^1^-NMR spectroscopy and mass spectrometry provides quantitative analysis of metabolite concentrations and reproducible information with minimal sample handling. Puchades-Carrasco *et al*.^[35]^ performed ^1^-NMR spectroscopy metabolomic analyses of serum samples of MM patients and healthy subjects, and also compared the metabolic profiles of MM patients at the time of diagnosis and after achieving complete remission. Compared to healthy subjects, MM patients at the time of diagnosis exhibited higher levels of isoleucine, arginine, acetate, phenylalanine, and tyrosine and decreased levels of 3-hydroxybutyrate, lysine, glutamine, and some lipids. Interestingly, when myeloma patients achieved complete remission, the levels of lysine, citrate, lactate dehydrogenase (LDH), and choline went up whereas glucose level decreased in comparison to those at the time of diagnosis^[35]^.

A similar study using ^1^-NMR spectroscopy was performed by Ludwig *et al*.^[36]^ to characterize the metabolic profile within the bone marrow using filtered plasma derived from bone marrow aspirates of healthy donors and patients with MGUS or MM. This study found that the essential amino acids isoleucine and threonine were significantly decreased in the bone marrow of MGUS and MM patients as were the nucleotide-breakdown products, hypoxanthine and xanthine, suggesting an increase in anabolism. The products of arginine metabolism, creatinine and urea, were also significantly altered. The authors suggested that alterations in bone marrow metabolism are an early and consistent feature during the development of MGUS and MM^[36]^.

Recently, Gonsalves *et al*.^[37]^ performed ^13^C SIRM using U[^13^C_6_]-Glucose and U[^12^C_5_]-Glutamine in human MM cell lines to determine the contribution of carbon substrates from glutamine into the TCA cycle of clonal plasma cells in comparison with those from glucose. Their results suggest that glutamine is the main contributor of carbon substrates to the TCA cycle whereas glucose is an important contributor of carbon substrates for the formation of lactate in clonal plasma cells^[37]^. This was also supported by the finding that clonal bone marrow plasma cells in MM patients have higher glutamine uptake compared with the remainder of bone marrow mononuclear cells. Additionally, 2-hydroxyglutarate (2-HG) - an oncometabolite of glutaminolysis - is significantly increased in the bone marrow supernatant of MM patients compared to that of MGUS patients. Similarly, levels of 2-HG in the bone marrow supernatant or in the peripheral blood plasma of patients with smoldering multiple myeloma was associated with higher risk of progression to MM, and correlates with the percentage of bone marrow plasma cells expressing c-MYC^[37]^. These results demonstrate the utility of measuring 2-HG levels in BM supernatant or peripheral blood plasma of SMM patients as a potential biomarker for disease progression and for identification of patients who may benefit from early therapeutic intervention.

To characterize the lipid profile of MM cells, Hossen *et al*.^[38]^ sorted single MM and normal plasma cells using flow cytometry and performed matrix-assisted laser desorption/ionization-imaging mass spectrometry. They found that PC (16:0/20:4) was significantly decreased in MM cells compared to normal plasma cells, which is likely due to the decrease of its integrated fatty acids or to increased metabolism of C_16_:_0_.

The development of drug resistance is also associated with metabolic alterations in MM. Zub *et al*.^[39]^ performed global proteomic and transcriptomic profiling on melphalan sensitive and resistant RPMI8226 MM cell lines. Glycolysis was found to be the most significantly altered pathway. Gluconeogenesis, glutaryl- CoA degradation, isoleucine and tryptophan degradation, TCA cycle, ketolysis and ketogenesis were also significantly altered when MM cells develop resistance to melphalan. Specifically, the glycolytic and pentose phosphate pathway enzymes were up-regulated in the melphalan resistant cells, whereas the TCA cycle and ETC proteins were down-regulated. Additionally, the melphalan-resistant cells displayed increased tolerance to overall oxidative stress, but were sensitive to mitochondrial electron transport inhibitors. It was further demonstrated that lactate accumulation, Interleukin-8 and vascular endothelial growth factor (VEGF) signaling, as well as aldo-keto reductase played a role in the development of drug resistance to melphalan in MM cell lines^[39]^. Similarly, in MM cell lines resistant to proteasome inhibitors (bortezomib and carfilzomib), complex proteomic changes, particularly in redox and energy metabolism were observed^[40]^. Proteins involved in metabolic regulation, redox homeostasis, and protein folding and destruction were upregulated in proteasome inhibitor-resistant MM cell lines, which exhibited metabolic adaptations that favored the generation of reducing equivalents such as NADPH^[40]^. The glucose metabolism was rewired in bortezomib-resistant MM cells, leading to higher activity of both the pentose phosphate pathway and serine synthesis pathway and an increased anti-oxidant capacity in bortezomib-resistant cells^[41]^. Interestingly, the expression of 3-phosphoglycerate dehydrogenase (PHGDH), which catalyzes the rate-limiting step of serine synthesis, was up-regulated in bortezomib-resistant MM cells. Consistent with increased serine synthesis observed in bortezomib-resistant MM cells, serine starvation enhances the cytotoxicity of bortezomib. These data suggest that interfering with serine metabolism could be a novel strategy to improve bortezomib therapy and PHGDH could be a potential biomarker for bortezomib resistance^[41]^.

To identify therapeutic targets for the prevention and treatment of drug resistance, Maiso *etal*.^[42]^ performed metabolic profiling of multiple myeloma cells in normoxic and hypoxic conditions. It was found that in hypoxic conditions, intermediates of the TCA cycle and the ETC were reduced whereas intermediates of glycolysis were elevated. Specifically, glucose 6-phosphate, fructose 6-phosphate, fructose 1,6-bisphosphate, pyruvate, and lactate were significantly increased in hypoxic cells. Gene expression profiling further demonstrated that the main pathways altered by hypoxic culture were those involved in glucose metabolism and the TCA cycle including key metabolic genes such as hexokinase 2 (HK2) and lactate dehydrogenase A (LDHA). Interestingly, bortezomib treatment inhibited HK2 activity but not lactate activity, suggesting LDHA may play a role in modulating drug resistance of MM cells in hypoxic conditions.

## TARGETING METABOLIC PATHWAYS FOR THE TREATMENT OF MULTIPLE MYELOMA

The alterations in metabolism provide potentially therapeutic targets for the treatment of MM.

### Targeting glycolysis for the treatment of MM

Glucose is the most abundant energy producing molecule in the human body and as such is broken down to produce energy for the body to maintain its regulatory functions. MM exhibits significant alterations in glucose metabolism. This was demonstrated by the fact that myelomatous bone and soft tissue lesions have elevated glucose uptake on positron emission tomography (PET) scans. Total lesion glycolysis and metabolic tumor volume, parameters measurable using ^18^F-FDG PET CT scan, are highly associated with progressionfree and overall survival and can significantly improve the prognostic value of both the GEP70 and International Staging Systems^[43]^. These data suggest that targeting specific aspects of glucose metabolism may offer a novel avenue for therapy.

Like many other tumors, MM cells demonstrate enhanced glycolysis and lactate production (e.g., aerobic glycolysis) instead of proceeding through the TCA cycle. Enhanced glycolytic flux confers tumor cells a growth advantage and plays an important role in maintaining myeloma cell survival and proliferation and in inducing chemoresistance. Many genes are involved in the enhanced glycolysis seen in MM. The PI3K/AKT pathway, a cytoplasmic chemical messaging system, has been linked to increased glucose metabolism and may be part of the reason why glycolytic intermediates are upregulated in myeloma cells^[44-47]^. Subsequently, it was found that a serine/threonine protein kinase, mTOR, regulated PI3K/AKT signaling^[45]^. Hypoxia-inducible factor-1 (HIF-1) is a transcription factor that is upregulated in the bone marrow microenvironment (where hypoxic conditions are standard), in myeloma cell lines, and CD138+ plasma cells isolated from MM patients^[48,49]^. HIF-1 has been shown to play a key role in the accumulation of increased glycolytic metabolites in MM^[42,50,51]^. HIF1-α (a subunit of HIF-1) induces transcription of several genes related to the response to hypoxia including genes that upregulate glycolytic enzymes and lactate production^[42]^. HIF1-α expression is increased in drug resistant myeloma cells, and is associated with increased risk for metastatic disease and worse prognosis in various cancers^[42,50-53]^. Drug resistance and disease relapse are thought to be due to the minimal residual disease cells that reside in the hypoxic bone marrow microenvironment. Maiso *et al*.^[42]^ showed that specific inhibition of LDHA and HIF1-a can restore drug sensitivity to anti-myeloma agents and inhibit tumor growth suggesting that targeting HIF1-a or LDHA can be used to inhibit myeloma growth and overcome drug resistance.

Pyruvate kinase M2 (PKM2) is a key factor regulating aerobic glycolysis and promoting tumor cell proliferation and survival^[54]^. Recently, Gu *et al*.^[55]^ showed that never in mitosis gene A (NIMA)-related kinase 2 (NEK2) regulates splicing of PKM and increased the PKM2/PKM1 ratio in myeloma cells to promote aerobic glycolysis and oncogenic activity.

LDH is a key enzyme that regulates glycolysis and the conversion of pyruvate and NADH to lactate and NAD^+^ respectively^[56]^. LDHA has been shown to be upregulated in MM cells by the proliferator-activator receptor-g coactivator-1b acting on the LDHA promoter. This increase has been shown to significantly potentiate glycolysis metabolism resulting in increased cell proliferation and tumor growth^[42,57]^.

MM cells exhibit increased activity in glucose transporters and in key glycolytic enzymes such as HK. Most of the work targeting glucose metabolism in MM has focused on the GLUT family. Up-regulation of GLUT1, GLUT4, GLUT8, and GLUT11 has been shown to increase glycolytic metabolites in myeloma cells^[58]^. Myeloma cells rely on the insulin-responsive glucose transporter GLUT4 for basal glucose consumption, maintenance of Mcl-1 expression, growth, and survival^[58]^. Ritonavir is an FDA-approved HIV protease inhibitor and has a selective off-target inhibitory effect on GLUT4. Treatment with ritonavir elicits dose-dependent abrogation of both glucose uptake and proliferation on KMS11 and L363 myeloma cells^[58]^. Interestingly, a subset of myeloma cells survives glucose deprivation or ritonavir treatment likely by engagement of mitochondrial oxidative phosphorylation. Metformin is an FDA approved anti-diabetic medication that targets mitochondrial complex 1. The combination of ritonavir and metformin effectively elicited apoptosis *in vitro* in MM cell lines and primary human myeloma cells and showed anti-myeloma activity *in vivo* in a xenograft model of MM^[59]^. Further analysis of the combination showed that it suppressed AKT and mTORC1 phosphorylation, and down-regulated the expression of Mcl-1 in myeloma models^[59]^.

Hexokinases catalyze the first irreversible step of glycolysis and play a critical role in the regulation of glycolytic activity. HK2 interacts with the voltage dependent anion channel in the outer membrane of mitochondria, where it catalyzes the conversion of glucose to glucose 6-phosphate^[60]^. HK2 has been shown to be overexpressed in a variety of cancers including MM^[61,62]^, suggesting that HK2 could be a viable target for inhibiting the proliferation of multiple myeloma cells^[62-65]^. Notably, treatment with bortezomib or vincristine, downregulated the expression of GLUT-1 and hexokinase, and induced apoptosis in OPM2 MM cells^[66]^.

An additional approach to targeting glucose consumption has been with the use of the novel purine analogue 8-aminoadenosine (8-NH(2)-Ado). Shanmugam *et al*.^[67]^ showed that treatment of the MM1S myeloma cell line with 8-NH(2)-Ado reduced glucose consumption through regulation of the GLUT4 transporter.

### Targeting amino acid metabolism for the treatment of MM

In addition to MM cells being dependent on glucose, myeloma cells are also dependent on glutamine for energy equivalents through a process known as glutaminolysis. This process is especially prevalent in chemotherapy resistant myeloma cells. Bajpai *et al*.^[68]^ showed that when glucose metabolism is inhibited in myeloma cells exposed to various anti-myeloma agents, (bortezomib, melphalan, or carfilzomib), the resistant myeloma cells became preferentially dependent on glutamine and thus less likely to undergo apoptosis. Using glutamine as a primary energy source also affects the bone marrow microenvironment in MM patients. Specifically, the T-cells and NK cells in the tumor microenvironment are suppressed by the nutrient deprivation, hypoxia, and decreased pH that results from increased dependence on glutamine as an energy source^[69]^.

Glutaminolysis breaks down glutamine into alpha-ketoglutarate which then enters TCA cycle. Glutamine can be converted to glutamate, a reaction termed glutamine anaplerosis. Alpha-ketoglutarate can be further broken down to 2-HG which is associated with c-MYC overexpression^[37,70]^. 2-HG is an oncometabolite, and elevated levels of 2-HG in smoldering MM patients are associated with increased risk of progression to MM^[37]^. Glutamine is crucial for the survival and proliferation of certain cancer cells, and starvation of glutamine induces cancer cell death^[71]^. Effenberger *et al*.^[72]^ recently showed that *in vitro* cultures of MM cells are dependent on glutamine for survival and that this is dependent on MYC protein expression. Notably, when these cells were treated with the glutaminase inhibitor benzophenanthridinone 968 apoptosis was induced. Additionally, MM cells show high expression of the glutamine transporters SNAT1, ASCT2, and L-type amino acid transporter 1 (LAT1); and inhibition of the ASCT2 transporter exhibits anti-myeloma activity^[73]^. Furthermore, high expression of LAT1 is associated with high proliferation and poor prognosis in newly diagnosed MM patients, and predicts poor overall survival independent of the International Staging System^[74]^. These data demonstrate that inhibition of glutaminolysis serves as a potential therapeutic approach in the treatment of MM^[71,72]^.

### Targeting fatty acid metabolism for the treatment of MM

Fatty acids and lipids play important roles in the development and pathogenesis of MM through direct influence on the metabolism of myeloma cells, functioning as intercellular messengers, or acting as mediators of the pathological immunologic pathways^[75]^. Analysis of plasma or erythrocyte membrane lipid composition provides a simple, suitable model to study fatty acid metabolism. Jurczyszyn *et al*.^[75]^ measured the fatty acid composition of RBC membranes in MM patients and compared them with healthy controls. MM patients exhibited higher levels of saturated fatty acids and n-6 polyunsaturated fatty acids (PUFA) and lower levels of monounsaturated n-3 PUFA and trans-fatty acids, than controls. It was suggested that the fatty acid content of the RBC membrane could serve as a diagnostic and/or predictive biomarker in MM. Similarly, increased plasma levels of saturated fatty acids and n-6 PUFA were observed in MM patients compared to healthy controls, indicating increased synthesis of these fatty acids in MM patients^[76]^. Recently, it has been shown that several myeloma cell lines (U266, RPMI8226, and NCI-H929) increase fatty acid oxidation as a mechanism to maintain metabolic hemostasis and viability during conditions of decreased glycolysis and increased lactate accumulation^[77-79]^.

Fatty acid synthase (FAS) expression was up-regulated in human myeloma cell lines as well as primary myeloma cells and contributes to myeloma cell proliferation and survival. Inhibition of fatty acid β-oxidation with etomoxir or *de novo* fatty acid synthesis with orlistat significantly reduced myeloma cell proliferation. The combination of 50 mmol/L etomoxir and 20 mmol/L orlistat resulted in an additive inhibitory effect on myeloma cell proliferation. Interestingly, the inhibitory effect was associated with reduced levels of p21 protein and phosphorylated retinoblastoma protein^[79]^. These data suggest that inhibition of fatty acid metabolism provides a potential therapeutic approach in the treatment of MM, however O’Connor *et al*.^[80]^ recently showed that etomoxir loses its specificity at concentrations above 5 mmol/L and results in increased ROS production so these experiments must be interpreted with caution.

Obesity is associated with increased risks of myeloma incidence and mortality. To understand the molecular mechanisms by which adipocytes contribute to myeloma cell survival and progression, Bullwinkle *et al*.^[81]^ co-cultured MM cell lines with adipocytes harvested from normal, overweight, obese, or super obese individuals. It was found that MM cells proliferated faster, displayed increased pSTAT-3/STAT-3 signaling, and had enhanced adipocyte endothelial tube formation and cell adhesion when co-culturing with conditioned media from obese and super obese individuals.

## FUTURE DIRECTIONS

Several areas of future research provide promise in the development of novel treatments for MM.

### Lactate research

As the primary molecule thought to induce the hypoxic microenvironment surrounding many cancer cells, lactate is one of the most heavily researched metabolites in MM cells. In 1991 it was discovered that high serum LDH levels could identify myeloma patients that were at increased risk for early progression after treatment^[82]^. Furthermore, LDH has been identified as a marker that identifies higher risk patients^[83]^. The increased anaerobic respiration in the hypoxic microenvironment of myeloma cells is thought to be linked to the HIF1-a transcription factor, and leads to an increase in lactic acid production. In MM, HIF1-a activity increases glycolytic metabolites, inhibits production of TCA intermediates, and activates IL-6 which all contribute to tumor growth and survival^[84,85]^. Notably, hypoxia has been shown to promote myeloma cell dissemination from the bone marrow to the peripheral blood through the downregulation of E-cadherin and the upregulation of the epithelial to mesenchymal transition proteins SNAIL, FOXC2, and TGFβ1^[86]^. Hypoxic conditions also promote upregulation of VEGF leading to increased angiogenesis in a mechanism that is thought to be related to HIF1-a activation^[86-89]^.

Transporters of lactate are also possible metabolic targets in myeloma. The MCT family of transporters are involved in lactate transport in and out of the cell. MCT1 and MCT4 are preferentially expressed in myeloma cells. Inhibition of MCT1 with α-cyano-4-hydroxycinnamic acid led to reduced lactate incorporation and induced apoptosis in myeloma cell lines^[90,91]^. Increased lactate is secreted out of MM cells through the MCT transporters leading to an acidification of the bone marrow microenvironment. This results in inhibition of MCTs in T-cells and thus accumulation of intracellular lactate and H+, which-together with their reduced access to glucose by overconsuming tumor cells-leads to decreased T-cell activation^[92]^.

### OXPHOS in multiple myeloma

Since oxidative phosphorylation is the last step in ATP synthesis for aerobic respiration it has been a particular focus of research in cancer metabolism. Given the increase in glycolysis during the progression of myeloma reviewed above, it might be predicted that OXPHOS increases as well. However, it has been found that OXPHOS activity is decreased during increased glycolysis in MM. This is thought to occur because the TCA cycle is not producing enough electron carriers to supply the OXPHOS mechanism to operate at a normal rate of ATP production^[90]^. Given these conditions, energy production shifts to anaerobic respiration since other mechanisms, like ketosis, are evolutionarily reserved for starvation and extreme cases, meaning that lactate is the main source of energy in myeloma cells.

While many groups have investigated the role of metabolism in myeloma, relatively few have focused on the role of oxidative phosphorylation in MM. The complex 1 inhibitor, IACS-010759, has been tested in leukemia cells where it led to decreased proliferation and increased apoptosis in leukemia cells^[93,94]^. This compound is actively being tested in clinical trials of relapsed AML (NCT02882321), and advanced solid tumors and lymphomas (NCT03291938).

Similarly, inhibitors of complex III have been explored as possible anti-cancer agents. Inhibition of complex III leads to an increase in ROS which can combat tumor proliferation and metastasis as well as induce apoptosis in MM. Previously, Arihara *et al*.^[95]^ demonstrated that reactive oxygen species have antimyeloma activity. Using CP-31398, an activator of p53 which also induces the formation of ROS, the investigators demonstrated decreased MM proliferation and increased apoptosis in a p53 independent fashion, and this effect was synergistic with carfilzomib. Notably, no additional hematologic toxicity was seen with the agent in animal studies^[96]^.

### Fatty acid research

It is established that resistant MM cells can increase lipogenesis as a mechanism of chemotherapy resistance^[42,79,97]^. PUFAs have previously been shown to enhance chemotherapeutic drug effects by selectively inducing apoptosis in multiple cancer cell lines^[98-103]^. Specifically, administration of two PUFAs, eicosapentaenoic acid (EPA) and docosahexaenoic acid (DHA) to four MM cells (L363, OPM-1, OPM-2 and U266) resulted in induction of apoptosis through increased caspase-3 activation, and mitochondrial membrane perturbation with no effect on normal human peripheral mononuclear cells^[104]^. Similarly, studies by Dai *et al*.^[105]^ examined the effects of EPA and DHA on the myeloma cell lines MM1S and MM1R and found that treatment with these agents resulted in increased apoptosis which was enhanced by the addition of dexamethasone. Notably, this synergy with dexamethasone was observed in the MM1R cell line which is inherently dexamethasone resistant. As glucocorticoids are known to increase lipogenesis, and remain a mainstay of MM therapy, these data suggest that EPA and DHA may resensitize cells that have acquired resistance to glucocorticoids. Additional studies on MM cell lines showed that treatment with EPA or DHA decreased MM cell proliferation through induction of lipid peroxidation. This effect was inhibited by superoxide dismutase, or cyclooxygenase/lipooxygenase inhibitors suggesting a role for superoxides, prostaglandins, and leukotrienes in MM proliferation^[106]^. Similarly, Wang *et al*.^[107]^ examined the expression of FAS in bone marrow from MM patients and matched healthy volunteers and found 70% of MM patients had elevated FAS while none of the healthy volunteers had detectable levels. Treatment of the FAS expressing MM cell lines U266 and RPMI8226 with the FAS inhibitor cerulenin resulted in induction of apoptosis evidenced by increased Annexin V staining suggesting FAS as a possible target of anti-myeloma therapy. These studies are admittedly preliminary but warrant additional research in this area.

### Other metabolic targets

Other studies have been focused on TCA intermediates. Sanchez *et al*.^[108]^ were able to identify dichloroacetate (DCA) as a possible target for cancer therapy. DCA is an activator for pyruvate dehydrogenase which allows for increased production of TCA intermediates and consequently increased OXPHOS for ATP production. Subsequently DCA was found to inhibit glycolysis in multiple myeloma and increase sensitivity to bortezomib.

## CONCLUSION

The study of metabolism’s role in MM remains in its infancy and many avenues remain unexplored. With combination therapies being the norm in myeloma management and acquired resistance to conventional therapies remaining a challenge, the potential exists for additional adjunctive therapies in MM patients. Targeting metabolic pathways is a novel area with preclinical data suggesting efficacy. Targets such as the GLUT and MCT transporters, IGF-1, FAS, and metabolites of the ETC and OXPHOS warrant additional exploration as possible novel anti-myeloma strategies, but care must be taken to minimize adverse effects when targeting ubiquitous pathways. Ideally, future preclinical and clinical studies will help to elucidate metabolism’s role in myeloma development and progression, and may lead to the discovery of novel therapies for patients suffering from this disorder.
